# Pseudo-chemotaxis of active Brownian particles competing for food

**DOI:** 10.1371/journal.pone.0230873

**Published:** 2020-04-08

**Authors:** Holger Merlitz, Hidde D. Vuijk, René Wittmann, Abhinav Sharma, Jens-Uwe Sommer

**Affiliations:** 1 Institut Theorie der Polymere, Leibniz-Institut für Polymerforschung Dresden, Dresden, Germany; 2 Institut für Theoretische Physik II, Weiche Materie, Heinrich-Heine-Universität Düsseldorf, Düsseldorf, Germany; 3 Technische Universität Dresden, Institut für Theoretische Physik, Dresden, Germany; Pavol Jozef Safarik University in Kosice, SLOVAKIA

## Abstract

Active Brownian particles (ABPs) are physical models for motility in simple life forms and easily studied in simulations. An open question is to what extent an increase of activity by a gradient of fuel, or food in living systems, results in an evolutionary advantage of actively moving systems such as ABPs over non-motile systems, which rely on thermal diffusion only. It is an established fact that within confined systems in a stationary state, the activity of ABPs generates density profiles that are enhanced in regions of low activity, which is thus referred to as ‘anti-chemotaxis’. This would suggest that a rather complex sensoric subsystem and information processing is a precondition to recognize and navigate towards a food source. We demonstrate in this work that in non-stationary setups, for instance as a result of short bursts of fuel/food, ABPs do in fact exhibit chemotactic behavior. In direct competition with inactive, but otherwise identical Brownian particles (BPs), the ABPs are shown to fetch a larger amount of food. We discuss this result based on simple physical arguments. From the biological perspective, the ability of primitive entities to move in direct response to the available amount of external energy would, even in absence of any sensoric devices, encompass an evolutionary advantage.

## Introduction

The ability to move is among the common features of living systems. Mobility opens the opportunity to escape from dangerous environments and to search for food sources to increase the intake of energy-rich substances, which enhance the metabolism, grant survival and eventually the chance for reproduction—the central theme of the game of evolution. It is not surprising that mobile life-forms are abundant on all scales including the simplest microscopic organisms. Prominent examples are white blood cells chasing intruders [1], sperm cells rushing toward the oocyte [2] or bacteria such as Escherichia coli searching for food [3].

Active motion involves an investment of energy which only pays off if the opportunity for an increased consumption of nutrients is involved. This is the philosophy behind the term *chemotaxis*, initially referred to any directed movement of bacteria towards, or away from, chemicals, regardless of the underlying mechanism [4, 5]. Freely swimming bacteria often achieve chemotaxis by altering their tumble frequency, depending on temporal comparisons of stimulus intensity (i.e. nutrient concentration), a mechanism referred to as *kliokinesis with adaption* [4]. In this case, if turns are suppressed when organisms move up a gradient, they will eventually accumulate near the top of that gradient. Kliokinesis with adaption involves a delicate apparatus of chemical sensors and information processing and is therefore restricted to organisms of advanced level of evolution. If the tumble rate changes only locally with stimulus intensity, i.e. without the help of temporal comparisons, the so called *kliokinesis without adaption* results in a uniform stationary distribution of the organisms in which chemotaxis is absent. In contrast, the underlying mechanism of *orthokinesis* referres to a response of the swim speed to the local food concentration. An increase of speed at high food concentrations makes the organisms accumulate where activity is low, commonly known as *reverse chemotaxis* [6] or *anti-chemotaxis* [7]. Both mechanisms, kliokinesis (without adaption) and orthokinesis are summarized under the term *chemokinesis* [8].

As a matter of fact, a precursor to the stationary distributions associated with chemokinesis may—under certain conditions—feature transient states in which the bacteria actually run up the nutrition gradient to exhibit a kind of temporary chemotactical behavior. This phenomenon, that takes place in absence of any temporal comparisons of nutrient concentrations and may thus be accessible to rather primitive organisms, has been named *pseudo-chemotaxis* [9]. It is important to note that the underlying system setup is anything else but uncommon in daily life situations: While stationarity relies on small, closed compartments inside which the organisms reach equilibrium distributions under a constant flow of nutrients, a transient behavior is common in open systems in which nutrients pop up randomly and temporarily while organisms compete for limited supplies. Whether or not pseudo-chemotaxis enables primitive entities to score an advantage in terms of nutrient consumption will be the theme of the present simulation study.

As our model for orthokinetic organisms serve ABPs that respond to local concentrations of chemical supplements to the solvent, featuring a mechanism that leads to a self-propulsion of the particles [10, 11]. ABPs obtain motility by consuming energy, e.g. in the framework of catalytic reactions with supplements such as hydrogen peroxide [12] or hydrazine [13] to the solvent, or through photo-activated self-diffusiophoresis [14]. The resulting motion is persistent but pointing into an arbitrary direction in the laboratory frame. Due to diffusive rotation, the direction of motion is randomized after a characteristic time, hence the term *Brownian*. In order to study chemotaxis, a gradient of activity—which can be understood as a gradient in ‘food’ or ‘fuel’ concentration—is implemented. Pseudo-chemotaxis of ABPs has recently been observed in computer simulations and analyzed in terms of theoretical models [15–17]. The corresponding coarse-grained Fokker-Planck equation has been shown to exhibit a macroscopic flux term that is absent with passive BPs [17]. We point out that another mechanism which could induce drifts of particles inside concentration gradients, the *down-gradient diffusiophoresis* [18], is of no relevance in the present study since particles in our model setup are lacking excluded volume interactions.

In the laboratory, synthetic self-propelling agents, notably Janus-particles of spherical shape, are designed and employed to study the features of simple self-propelling motors. The smallest Janus-particles are of dimensions of only 30nm and thus of the same order as some of the proteins found in living systems [19]. As a matter of fact, catalytic enzymes have been reported to exhibit an enhanced diffusion during catalytic activity [20–22], and the gain in mobility has recently been claimed to originate from a (so far unknown) mechanism of self-propulsion [7, 23]. The presence of orthokinesis in catalytic enzymes would imply that there actually exist biological systems, far simpler than living cells or bacteria, which feature a self-propelled motion on a similarly low level of complexity as synthetic Janus-particles.

In the present work, we pick up previous studies of ABPs in activity gradients [17] and—motivated by biological evolution—attempt to approach the phenomenon of pseudo-chemotaxis from another angle: While transport properties such as first passage times and target hit probabilities have been studied before in the presence of pre-defined background activity fields [16, 17], the present work interprets activity as a result of the intake of explicit food (or fuel) particles. Two species, ABPs, which temporarily turn active after food intake, and otherwise identical BPs, which consume food but remain inactive, are competing for a limited amount of food available in the system. Contrary to previous studies, the particles thus modify their environment through the consumption of fuel and create a rather competitive ‘first come, first served’ setup. The choices for the particle properties (e.g. diameter of 30nm, propulsion force of 1pN, see Table 1) are of the same order as those encountered in actual experiments [19, 23]. In order to help bridging the two worlds of experiment and theoretical non-equilibrium statistical mechanics, and to make this work accessible to a wide range of scientists, including biologists, we lay focus on phenomenology and dispense with a formal mathematical modeling, instead referring to the existing literature.

## Materials and methods

**Fig 1:**
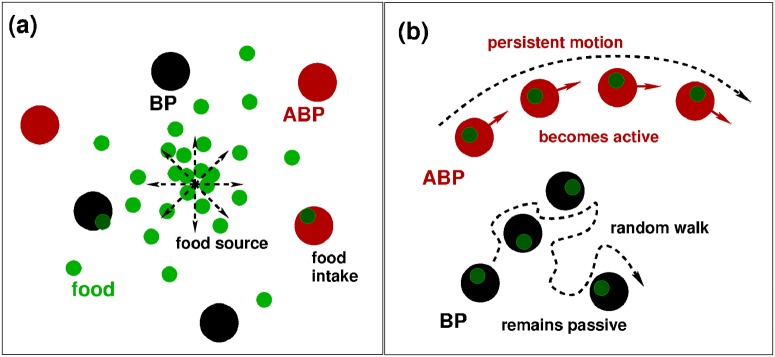
System setup. Left: The system consists of a food source that emits food particles (green), surrounded by BPs (black) and ABPs (red). Right: If an ABP runs into a food particle, it temporarily turns active and exhibits a persistent motion. The BP consumes food, too, but remains a passive Brownian walker. Which strategy is superior?

Two types of particles exist (see Fig 1): First, the standard BPs and, second, self-propelling ABPs, activated for a given time-period, called boost time *τ*_B_, after consuming a dimensionless (point-like) food particle. The spherical particles have diameters of *b* = 30nm and are dispersed in an implicit solvent of temperature *T* = 298K and dynamic viscosity *η* = 0.89mPa⋅s (corresponding to water at 298K). The resulting passive (i.e. in absence of self-propulsion) translational diffusion coefficient amounts to *D*_t_ = 0.016nm^2^/ns, and the rotational relaxation time is of the order of *τ*_r_ = 9.2 · 10^3^ns.

ABPs—when active—are driven with a force of *f* = 1pN, directed along the axis of their current orientation. A driven particle exhibits—on time scales that are large compared to its rotational relaxation time—a motion that resembles Brownian motion with an increased effective diffusion coefficient [24]

$$\begin{array}{c} D_{\text{eff}}=D_{t}+D_{a}=\frac{k_{B}T}{\zeta }+\frac{f^{2}\tau_{r}}{\zeta^{2}d} \end{array}$$ (1)

with the frictional drag coefficient *ζ* = 3*πηb* and the spacial dimension *d* = 3. Here, the active contribution to the diffusion coefficient amounts to *D*_a_ = 0.048nm^2^/ns, thrice the value of the passive diffusion. The driving force of 1pN is of the same order as recently proposed forces acting on catalytic enzymes of similar sizes [23]. We assume that the activity does not affect the rotational diffusion of the ABP.

The point-like food particles are assumed to move with a translational diffusion coefficient that is larger by a factor five when compared to the BPs. A food particle is ‘consumed’, i.e. removed from the system, as soon as it occupies the space taken by another particle.

Both BPs and ABPs may consume an unlimited number of food particles at any time (being active or not). After consumption of each food particle, the ABPs remain active only for a fixed time period, called boost time *τ*_B_, i.e. no accumulation and storage of energy beyond a single quantity is taken into account. Urease has been measured to display an increased diffusivity over time periods of roughly 10*μ*s [23], and this is the order of magnitude of the boost time we have chosen for our ABPs.

Since we are interested in the dynamics of single particles, pair-interactions between particles as well as hydrodynamic interactions are absent. In confined systems, all particles (including food) interact with the confining walls via a standard short-range repulsive Weeks-Chandler-Andersen-potential [25].

The time integration is carried out using a standard second-order Brownian dynamics algorithm [26]. A tentative first-order displacement is:

$$\begin{array}{c} r_{i}^{\prime }\left(t+dt\right)-r_{i}\left(t\right)=\frac{F_{i}\left(r,t\right)}{\zeta }\;dt+\gamma_{i}\left(t\right)\;,\;\;\;i=1,\ldots ,N \end{array}$$ (2)

Here, the total force on the ith particle, ***F***_*i*_, is the sum of all conserved forces (from boundary walls if present) and, in case of ABPs, the driving force

$$\begin{array}{c} f_{i}\left(t\right)=f_{i}\left(t\right)p_{i}\left(t\right)\;,\;\;\;i=1,\ldots ,N^{\prime } \end{array}$$ (3)

with the coordinates ***r***_*i*_, the forces *f*_*i*_(*t*) (temporarily applied during the boost-time *τ*_*B*_ after food-consumption) and the embedded unit orientation vectors ***p***_*i*_. *N* is the total number of particles, *N*′ the number of ABPs, *ζ* the frictional drag coefficient and *dt* the time step. The stochastic vectors ***γ***_*i*_(*t*) are Gaussian distributed with zero mean and time correlations $\langle \gamma_{i}\left(t\right)\;\gamma_{j}^{T}\left(t^{\prime }\right)\rangle =2D_{t}dt1\delta \left(t-t^{\prime }\right)\delta_{ij}$ with the identity matrix 1 and the translational diffusion coefficient *D*_*t*_. The second half-step is:

$$\begin{array}{ccc} r_{i}\left(t+dt\right)-r_{i}^{\prime }\left(t+dt\right) & = & \frac{-F_{i}\left(r,t\right)+F_{i}^{\prime }\left(r,t+dt\right)}{2\zeta }\;dt\;,\\ i & = & 1,\ldots ,N \end{array}$$ (4)

where $F_{i}^{\prime }\left(r,t+dt\right)$ are the forces calculated for the conformation $r_{i}^{\prime }\left(t+dt\right)$. Finally, the orientation vector is updated via

$$\begin{array}{c} p_{i}\left(t+dt\right)-p_{i}\left(t\right)=\eta_{i}\left(t\right)\times p_{i}\left(t\right)\;,\;\;\;i=1,\ldots ,N^{\prime } \end{array}$$ (5)

with the stochastic vectors ***η***_*i*_(*t*), which are Gaussian distributed with zero mean and time correlations $\langle \eta_{i}\left(t\right)\;\eta_{j}^{T}\left(t^{\prime }\right)\rangle =2D_{r}dt1\delta \left(t-t^{\prime }\right)\delta_{ij}$, and the rotational diffusion coefficient *D*_*r*_. Values for the simulation parameters are summarized in Table 1.

**Table 1:** Simulation parameters and derived quantities.

Quantity	Meaning
*d* = 3	dimension of space
*b* = 30 *nm*	particle diameter
*m* = 14.14 *ag*	particle mass
*dt* = 10 *ns*	simulation timestep
*T* = 298 *K*	system temperature
*f* = 1 *ag* ⋅ *nm*/*ns*^2^	driving force (ABP)
*η* = 0.981 *mPa* ⋅ *s*	viscosity (solvent)
*ζ* = 3*πηb* = 251.9 *ag*/*ns*	frictional drag coeff.
*τ*_*m*_ = *m*/*ζ* = 0.0561 *ns*	momentum relax. time
*D*_*t*_ = *k*_*B*_ *T*/*ζ* = 0.0163 *nm*^2^/*ns*	passive diffusion coeff.
*D*_*r*_ = 3*D*_*t*_/*b*^2^ = 5.45 ⋅ 10^−5^ *ns*^−1^	rot. diffusion coeff.
*τ*_*r*_ = 1/(2*D*_*r*_) = 9180 *ns*	rot. relaxation time
*v* = *f*/*ζ* = 3.97 ⋅ 10^−3^ *nm*/*ns*	final velocity (ABP)
*D*_*a*_ = *v*^2^ *τ*_*r*_/*d* = 0.0482 *nm*^2^/*ns*	activated diffusion coeff.
*T*_*a*_ = *ζD*_*a*_/*k*_*B*_ = 880 *K*	activated temperature

## Results

### Confined and stationary systems

**Fig 2:**
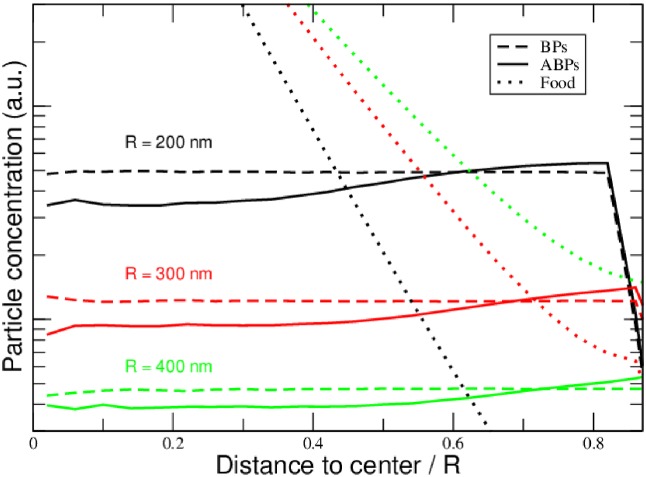
Particle densities in a stationary system. Particle concentration as a function of (relative) distance to the center, for different sizes *R* of the reactor. Different colors stand for different radii of the containers (black: 200nm; red: 300nm; green: 400nm). Close to the food source, ABPs are active most of the time, leading to a significant depletion of their concentration. Close to the outer wall (right edge of the graphic), particle concentrations drop due to steric repulsion. Food-production rate: 50/*μ*s; number of particles: 100 (of each species, ABPs and BPs); simulation time: 2s; boost time of ABPs: $\tau_{B}=2\tau_{r}\approx 18\mu \text{s}$.

In a first set of simulations, a total of 200 particles (100 of each species, BPs and ABPs) were placed into spherical confinements of radii *R* ∈ {200, 300, 400} nm. At the center, food particles were randomly generated at an average rate of 50 particles/*μ*s. After a sufficiently long simulation time, all particle distributions turned stationary and are displayed in Fig 2.

The stationary distributions of fuel-activated ABPs are non-uniform, being reduced in regions in which the food concentration is high (i.e. close to the center of the confinement). This is a consequence of the self-propulsion that comes along with their food consumption: It is well known that the stationary concentration of an ABP is inversely proportional to its average driven velocity and thus highest in regions of diminishing activity [6, 27]. In our simulations, this is visible at all system sizes, though the quantity of the ABP-depletion is highest in the small system (*R* = 200nm, solid black curve, note the logarithmic scale), while the distributions of BPs naturally remain unaffected by the food concentrations. The phenomenon observable in these simulations is thus the well known anti-chemotaxis of ABPs in stationary systems with activity gradients.

**Fig 3:**
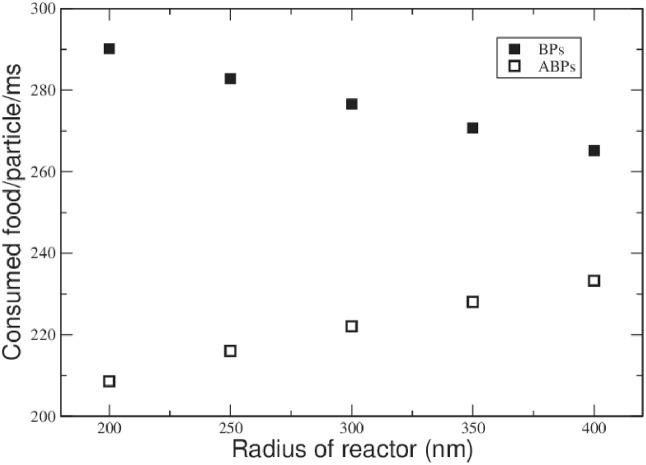
Stationary food consumption. Food consumption rates of particles for different sizes of the reactor. As a result of the bias of their density distributions, ABPs consume less food that BPs, exhibiting anti-chemotaxis. With increasing size of the reactor, the ratio of activated to inactive APBs is diminishing and their food consumption rates are approaching those of the BPs.

Fig 3 displays the average food consumption of each species in systems of different radii: In each case, the passive BPs are able to fetch food at a higher rate when compared to the ABPs, as a result of the reduced presence of ABPs close to the food source: As can be seen in Fig 2, the ABPs preferably occupy the peripheral region of the container in which nutrient concentration and hence ABP activity is low. The BPs assume a uniform concentration throughout the container, which exceeds the concentration of ABPs close to the center at which nutrient concentration is at its maximum. We have repeated the simulations with different parameter settings, varying boost times *τ*_B_ and food production rates, arriving at the same qualitative conclusions: Anti-chemotaxis reflects itself in the average food consumption of the active particle species, and any degree of activity may be regarded a disadvantage as soon as food is needed for the metabolism of a fictitious proto-life-form. However, the validity of this conclusion is restricted to the rather untypical scenario of a tightly confined stationary system, including a continuous supply of food, as is going to be demonstrated in the following section.

### Open and non-stationary systems

In the following set of simulations, we mimic a situation which may occur frequently in natural environments such as open bodies of water, e.g. ponds or lakes: A temporary food source emerges and several species in its vicinity start to compete for food. The system is unconfined (on relevant length-scales) and the distributions of different species are non-stationary. Once again, we compare the average food consumption of passive BPs and ABPs.

Sets of 100 representatives of each species are initially randomly distributed on a shell of distance *r*_ini_ to a food source, which emits a given number of (here: 5000) food particles in an instant. These food particles subsequently diffuse into all directions, run into the surrounding particles and are eventually consumed. The ABPs are activated for a boost time of $\tau_{B}=2\tau_{r}\approx 18\mu \text{s}$ upon consumption of a food particle, and this activity affects their transient concentration profiles.

**Fig 4:**
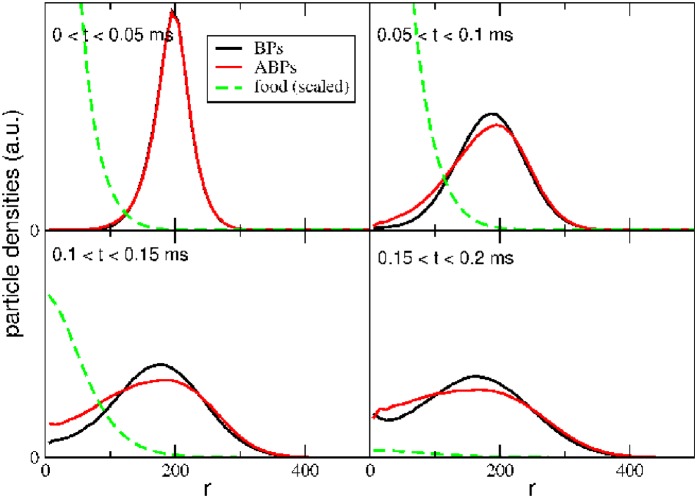
Transient density distributions. Time evolution of the density distributions of BPs (black) and ABPs (red), as well as food-particles (green, scaled down), for different time intervals. Initially, BPs and ABPs start at the distance *r*_ini_ = 200nm to the center, boundaries are absent. A burst of food particles occurs at the center at *t* = 0. Upon food consumption, the density distribution of ABPs advances rapidly toward the food source. Boost time of an ABP after consumption of a food-particle: $\tau_{B}=2\tau_{r}\approx 18\mu s$.

**Fig 5:**
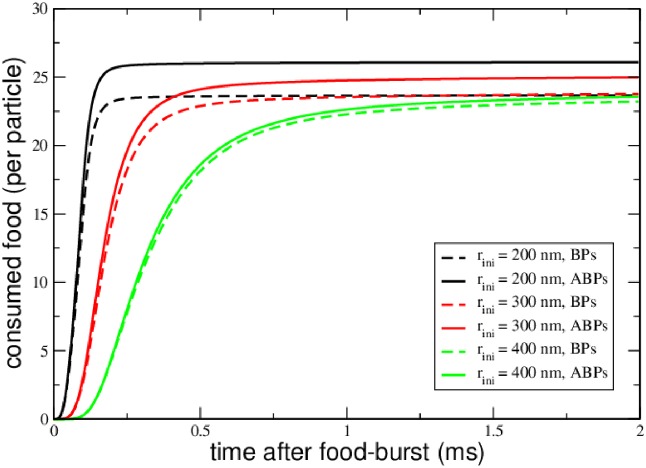
Food consumption in non-stationary setups. Consumed food particles as a function of time after begin of the food burst. As a result of the bias of their density distributions, ABPs consume more food than BPs, exhibiting chemotaxis. The differences diminish with increasing initial distance *r*_ini_ to the center, since fewer ABPs are activated.

The time evolution of these profiles is displayed in Fig 4 for the case of *r*_ini_ = 200nm, for different time intervals (chosen to keep the statistical noise low). The initial *δ*-distributions of both particle species begin to spread and to run into the expanding distribution of food particles (panel a). Motility, in the absence of external fields, can be mapped into a diffusion at elevated effective temperature [24] which accelerates the dispersion of density profiles in regions of activity. Consequently, the tail of the corresponding concentration profile of ABPs (red) reaches out toward the center of the food source at which food concentration is highest (panels b-d). When compared to the passive BPs (black profiles), the ABPs reach regions of high food concentration at an earlier time. This advantage is reflected in the rates of food consumption as shown in Fig 5 for three different initial distances of the particles: In particular at short distance to the food (black curves), the ABPs (solid curve) are able to fetch a larger number of food particles than the BPs (dashed curve).

The differences diminish with increasing initial distances of the particles (*r*_ini_ = 300nm, red curves, *r*_ini_ = 400nm, green curves), since here the food dilutes to a degree that the ABPs remain increasingly deactivated. If, however, the boost time *τ*_B_ is increased so that an ABP profits from elongated periods of activation, its advantage over the BP increases also in cases of larger initial distances to the food source (data not shown). In the supplementary part we also discuss simulations of systems with pure phases (either BPs or ABPs instead of mixtures). In Fig 2 and 3 of the supplementary part it is demonstrated how the ABP’s advantage in food collection over BPs almost diminishes in pure phases. This result supports the claim that it is the superior competitiveness of the ABPs that leads to the advantage shown in Fig 5.

In a non-stationary setup, ABPs are therefore exhibiting a kind of chemotaxis which enables them to consume a larger amount of food than their non-active competitors. We call this phenomenon *pseudo-chemotaxis* because it does not involve any sensing of food gradients on the part of the active particle. In fact, no orientation-bias is required for the emergence pseudo-chemotaxis, as was already shown in a previous work [17]: While ABPs do actually exhibit a small degree of orientation-bias inside activity gradients, this bias is pointing down the gradient and thus working against chemotaxis [28, 29]. Such an orientational polarization remains sufficiently weak to be negligible for the activity gradients considered here.

### The impact of food/fuel gradients

In order to analyze the importance of food concentration gradients, two test setups with immobile food particles are compared: First, the food has a Gaussian concentration: being allowed to diffuse from the location of their production for a short time and subsequently immobilized. In the second setup, food is distributed uniformly within a spherical volume of radius *r* = 100nm. Both setups contain 5000 food particles, while the remaining particle species start at *r*_ini_ = 200nm, outside the food sources, and the systems are unbounded. We are aware that the assumption of immobile food distributions is unrealistic, but here it serves as a pedagogical exercise to highlight the importance of gradients. One might imagine the nutrients being fixed inside gel-like structures which are permeable to ABPs.

**Fig 6:**
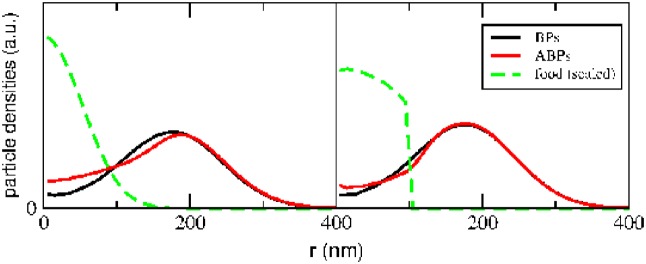
Transient density distributions. Time evolution of the density distributions of BPs (black) and ABPs (red), as well as food-particles (green, scaled down), averaged over the time interval of 0.1 < *t* < 0.15ms. Initial distance of the particles to the food source: *r*_ini_ = 200nm. Left panel: initial food profile was Gaussian, right panel: Initial food profile was a step-function. We remind that, despite the food being immobile, their profiles are depleted by the particles.

While the particle distributions spread, a certain fraction enters the food-reservoirs and begins to consume food. ABPs are then turning active and once again this activity affects the dynamics of their concentration profiles. Fig 6 displays typical snapshots of the concentration profiles, averaged over the time interval of 0.1 < *t* < 0.15ms after setoff. The system that features a food gradient (left panel) once again exhibits an increased concentration of ABPs close to the food source at which the food density is at its maximum. Contrary to that, the (initially) uniform food distribution leads to a different situation (right panel): Although close to the center of the food source, there exists an enrichment of ABPs compared to BPs, the situation is the opposite closer to the periphery of the food reservoir, at which BPs are more abundant than ABPs. Since the food distribution is at this moment still fairly uniform, the enrichment at the center does not encompass any significant advantage in terms of food intake.

**Fig 7:**
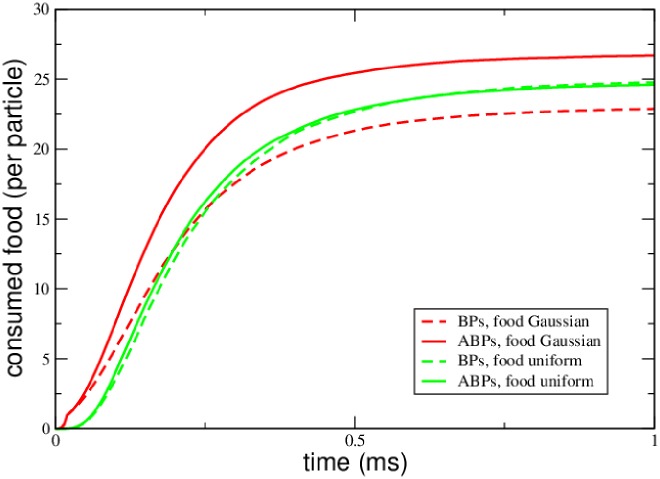
Non-stationary food consumption. Consumed food under the condition that the food concentration profile is a step function (green) or a Gaussian function (red). In the latter case, the ABPs experience an advantage over the passive BPs in terms of the amount of consumable food.

Fig 7 exhibits that both setups in fact lead to entirely different outcomes in terms of the food consumption: In the presence of a food gradient, the ABPs are able to fetch a higher number of food particles than passive BPs (red curves). Starting with a uniform food distribution does not lead to any systematic advantage for the ABPs (green curves). We have varied system parameters such a *r*_ini_ or the boost time *τ*_B_ of ABPs without arriving at qualitatively different outcomes: Whenever a gradient existed in the food concentration, the ABPs were able to take profit and gained a larger amount of food, while in the absence of such a gradient both BPs and ABPs received close to identical results. The phenomenon of pseudo-chemotaxis of ABPs in open, non-stationary systems is thus greatly supported by gradients which lead to high food/fuel concentrations.

## Conclusion

In the present work, we have approached the phenomenon of pseudo-chemotaxis of ABPs—recently discussed in the framework of transport properties such as first passage times and target hit probabilities [16, 17]—from another angle that is motivated by evolutionary considerations. The question that had to be addressed was: Does pseudo-chemotaxis actually lead to an advantage for a particle that is capable of chemokinesis, i.e. a food-induced enhancement of activity, yet unable to sense food gradients?

In our simulation study, two particle species had to compete for limited resources of food, the latter being simulated in terms of explicit food particles that could be consumed to trigger a temporary state of self-propulsion (in case of ABPs). The simulation parameters were chosen to be of similar dimensions as observed in real systems such as Janus particles of 30nm diameter [19] or catalytic enzymes, which appear to turn active during enzymatic activity [23].

The simulations confirm the well known fact that within a confined system and after reaching stationarity, the ABPs preferably occupy regions of low activity. Consequently, our fuel-activated ABPs do also consume a smaller amount of food than (otherwise identical) passive BPs. This is an unmistakable signature of anti-chemotaxis that has frequently been reported for ABPs in systems with activity gradients.

Non-stationary setups however can lead to the opposite situation in which ABPs exhibit an advantage over BPs. The term pseudo-chemotaxis has been coined because—contrary to ordinary chemotaxis – it does not involve any features related to sensing or information processing. The ABPs are running up activity gradients as a result of a purely statistical effect, related to their enhanced mobility in these regions, and the present work demonstrates that the observed bias in their transient concentration profile does in fact lead to an advantage over non-active competitors in terms of food of fuel intake. We have further demonstrated that not only the ability to turn active, but also the presence of a food concentration gradient – a rather common scenario in natural setups – was necessary for the ABPs to surpass the food consumption of their non-active competitors.

From a biological point of view it is certainly tempting to speculate about a potential relevance of these insights for the evolution of early proto-life-forms, which were sufficiently complex to feature a mechanism for self-propulsion, but yet unable to perform any kind of sensing or data processing. It would be astonishing if the feature of pseudo-chemotaxis would not have made it into the toolkits of early evolution. Catalytic enzymes may be modern examples for complex molecules which exhibit such a combination of features. Pseudo-chemotaxis might thus have been a vehicle—based entirely on non-equilibrium physical processes—which enabled similar entities to enter the game of evolution, to compete for fuel or food, the potential to enhance their metabolism, and eventually their reproduction rate.

As a next level of realism, food consumption could be coupled to the reproduction rates of species, while the energy required for self-propulsion could be accounted for. Pair-potentials between different species and food particles could be implemented to generate correlated dynamics and mimic predator-prey interactions, which might also include a minimalist model for information processing.

## Supporting information

S1 File (PDF)
