# Supplementary material for: Pseudo-chemotaxis of active Brownian particles competing for food
Source: PLoS One. 2020 Apr 8;15(4):e0230873. doi: 10.1371/journal.pone.0230873 (PMC7141648; doi:10.1371/journal.pone.0230873)
Supplement: S1 File — (PDF) [file pone.0230873.s001.pdf]

Pseudo-chemotaxis of active Brownian particles competing for food: Supplementary data

Holger Merlitz[1‡*], Hidde D. Vuijk[1◕], René Wittmann[2◕], Abhinav Sharma[1◕], Jens-Uwe Sommer[1,3◕]

1 Leibniz-Institut für Polymerforschung Dresden, Institut Theorie der Polymere, 01069 Dresden, Germany
2 Institut für Theoretische Physik II, Weiche Materie, Heinrich-Heine-Universität Düsseldorf, 40225 Düsseldorf, Germany
3 Technische Universität Dresden, Institut für Theoretische Physik, 01069 Dresden, Germany

‡This author wrote the manuscript. ◕These authors contributed equally to this work.
* merlitz@posteo.de

Simulations of pure BP and ABP systems

In the main text of the manuscript, all systems consist of mixtures of BPs and ABPs. This leads to a highly competitive environment in which nutrients are consumed by particles which arive first at its source. For comparison, this supplementary part is repeating some of the previous simulations with pure systems (BPs only vs. ABPs only).

Fig. 1 displays the stationary food concentration profiles in a closed system of radius $R = 200$nm with continuous food production. The black dashed curve belongs to the mixture of 100 BPs and 100 ABPs and is identical to the black dotted curve in Fig. 2 of the main text. In red is shown the food distribution corresponding to a pure system of 200 ABPs, in blue the food distribution in presence of 200 BPs. While BPs are uniformly distributed inside the system, the ABPs accumulate near the periphery at which the food concentration and hence activity is low. This distribution feeds back to the corresponding stationary food distribution which reaches somewhat further into the periphery of the container. To the contrary, the BPs, being of higher concentration in the central parts of the container, are able to deplete the nutrients rather efficiently so that the corresponding food concentration is lower when compared to the pure ABPs system or to the mixture. The total stationary food-intake rates are identical in all setups because the systems are closed and nutrition particles are unable to escape the container.

Fig. 2 displays the transient food concentrations in a setup that equals Fig. 4 in the main text, except for the purity of the BP phase (black) and ABP phase (red). At this early stage of the simulation, the food is depleted somewhat stronger by the ABPs, which - as a result of their increased diffusivity - reach the location of the food spill earlier than the BPs.

Fig. 3 contains the corresponding cumulated intakes of nutritients: While at earlier stages the pure ABP phase is capable of snatching food at higher rates, the BPs are catching up during later stages of the simulation. Since the nutrients are diffusing outwards and eventually running into the BPs, the BP's food consumption occurs later than the ABP's food intake, with little effect on the total number of consumed nutrients. This has to be contrasted to the situation in Fig. 5 of the main text (black

curves belonging to the same setup with $r_{\text{ini}} = 200\text{nm}$) in which both species compete for nutrients: There, the faster ABPs have consumed food which is consequently no longer available to the BPs. In the case of a direct competition between both species, the ABPs gain an advantage, which – in case of separate pure systems under identical conditions – is almost nonexistent.

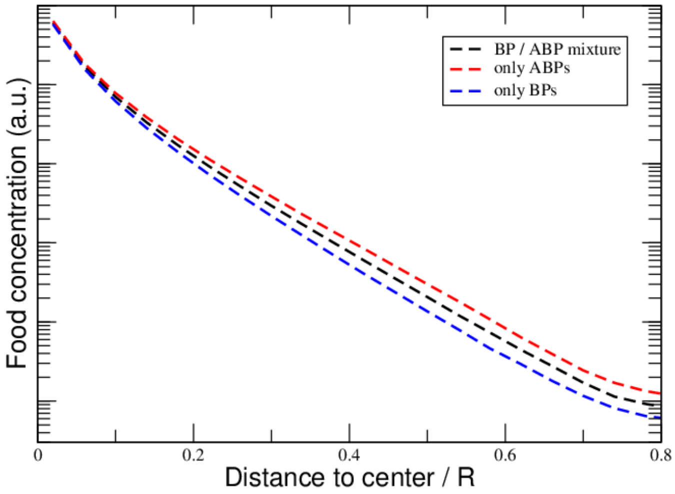

Fig 1. Particle densities in a stationary system. Stationary food concentration as a function of (relative) distance to the center, for a pure system of ABPs (red), a pure system of BPs (blue) and a symmetric mixture of both. Radius of the confinement: 200nm. Simulation parameters equal those of Fig. 2 in the main manuscript.

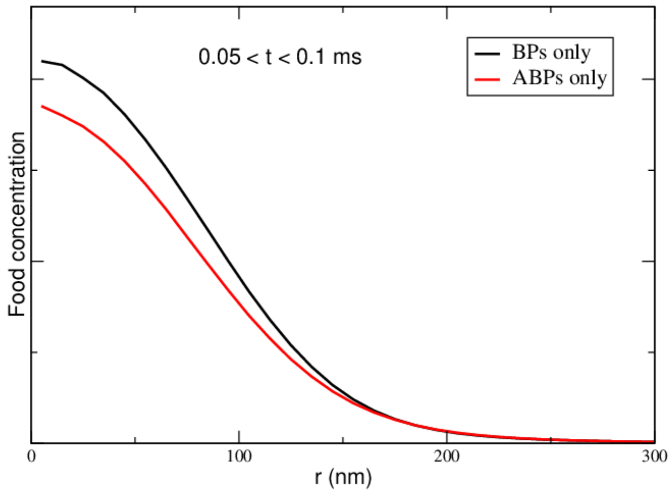

Fig 2. Transient food concentration in open system. Radial food concentration, averaged over the time interval 0.05ms $< t <$ 0.1ms after injection at $r = 0$. Black: in presence of 200 BPs; red: in presence of 200 ABPs. BPs and ABPs started at $t = 0$ at a distance of $r = 200$nm to the food source. Simulation parameters are equal to Fig. 4 in the main text.

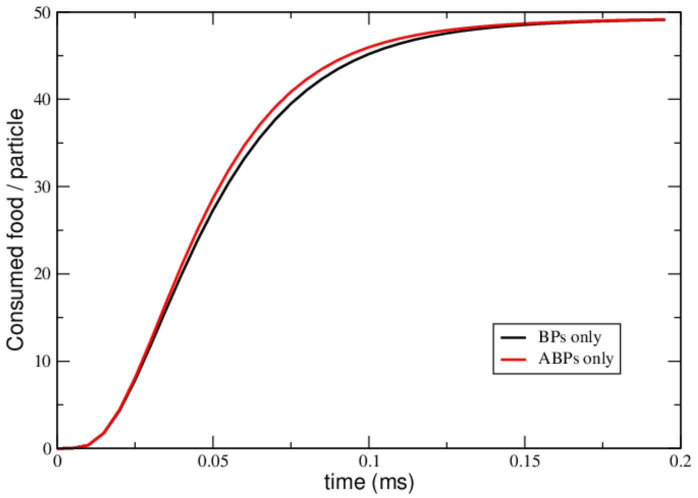

Fig 3. Food intake in case of a single burst. The accumulated number of consumed food particles (per BP or ABP) as a function of time after the food burst. BPs (black) and ABPs (red) start at $t = 0$ at $r_{\text{ini}} = 200$nm. The simulation parameters are identical to the case shown in Fig. 5 in the main text (black curves).
